# Reviewers BJCVS 31.3

**Published:** 2016

The Brazilian Journal of Cardiovascular Surgery (BJCVS) is grateful for the reviewers, listed below, which collaborated in this edition. Without their work would be impossible to keep the high scientific standard of our journal.

Carla Tanamati
Charles Simão Filho
Enio Buffolo
Francisco Fernandes Moreira Neto
Gilberto Venossi Barbosa
Giovani Jose Dal Poggetto Molinari
José Glauco Lobo Filho
Leonardo Andrade Mulinari
Lindemberg Da Mota Silveira Filho
Luciano Albuquerque
Luiz César Guarita Souza
Ricardo de Carvalho Lima
Rodrigo Milani
Rui M. S. Almeida
Tomas Salerno
Ulisses A. Croti
Victor Rodrigues Ribeiro Ferreira

